# First Report and Complete Genome Characterization of Cherry Virus A and Little Cherry Virus 1 from Russia

**DOI:** 10.3390/plants12183295

**Published:** 2023-09-18

**Authors:** Sergei Chirkov, Anna Sheveleva, Svetlana Tsygankova, Natalia Slobodova, Fedor Sharko, Kristina Petrova, Irina Mitrofanova

**Affiliations:** 1 Department of Virology, Faculty of Biology, Lomonosov Moscow State University, 119234 Moscow, Russia; anncsh@yandex.ru; 2 National Research Center “Kurchatov Institute”, 123182 Moscow, Russia; svetlana.tsygankova@gmail.com (S.T.); nv.slobodova@gmail.com (N.S.); fedosic@gmail.com (F.S.); petrova.k.o@yandex.ru (K.P.); 3 Faculty of Biology and Biotechnology, HSE University, 101000 Moscow, Russia; 4 Federal Research Center “Fundamentals of Biotechnology”, Russian Academy of Sciences, 119071 Moscow, Russia; 5 Research Center for Medical Genetics, 115552 Moscow, Russia; 6 Tsitsin Main Botanical Garden of Russian Academy of Sciences, 127276 Moscow, Russia; irimitrofanova@yandex.ru

**Keywords:** *Prunus* spp., virome, high-throughput sequencing, cherry virus A, little cherry virus 1, full-length genomes

## Abstract

Virus diseases affect the yield and fruit quality and shorten the productive life of stone fruits (*Prunus* spp. in the family *Rosaceae*). Of over fifty known viruses infecting these crops, cherry virus A (CVA) is among the most common, and little cherry virus 1 (LChV1) is one of the most economically important. Using high-throughput sequencing, full-length genomes of CVA and LChV1 isolates, found on interspecies hybrids in the *Prunus* collection of the Nikita Botanical Gardens, Russia, were sequenced, assembled, and characterized. CVA was found in the *P. cerasifera* × *P. armeniaca* hybrid and in phylogenetic analysis clustered with non-cherry virus isolates. The LChV1 isolate Stepnoe was detected in ((*P. cerasifera* Ehrh. × *P. armeniaca* L.) × *P. brigantiaca* Vill.) trihybrid suggesting that both *P. cerasifera* and *P. brigantiaca* potentially can be the LChV1 hosts. The isolate Stepnoe was most closely related to the Greece isolate G15_3 from sweet cherry, sharing 77.3% identity at the nucleotide level. Possibly, the highly divergent Russian isolate represents one more phylogroup of this virus. This is the first report of CVA and LChV1 from Russia, expanding the information on their geographical distribution and genetic diversity.

## 1. Introduction

All cultivated stone fruits (*Prunus* spp. in the family *Rosaceae*) are of great economic significance. Viruses affect the yield and fruit quality and shorten the productive life of the infected trees. The vegetative propagation that is widely used in the stone fruits cultivation contributes to the spread of viruses from plant to plant and into new regions with infected plant material. Over fifty viruses infecting these crops were discovered. In recent years, this list was continuously expanded due to the use of high-throughput sequencing (HTS) to study fruit tree viromes [1,2,3,4]. Cherry virus A (CVA) is among the most common *Prunus* viruses, and little cherry virus 1 (LChV1) is one of the most economically important [5,6]. 

CVA is a member of the genus *Capillovirus* in the family *Betaflexiviridae* [7]. The virus genome is single-stranded positive-sense polyadenylated RNA of about 7.5 kb with two open reading frames (ORFs). ORF1 encodes a polyprotein containing replication-associated proteins and the coat protein (CP). ORF2 is nested within ORF1 in a different reading frame and encodes the movement protein (MP). ORF1 is flanked with the 5′- and 3′-non-coding regions (NCR). 

CVA is widely distributed in cherry-growing regions worldwide and was also detected in non-cherry hosts such as peach (*P. persica*), apricot (*P. armeniaca*), plum (*P. domestica*), myrobalan (*P. cerasifera*), Japanese (Chinese) plum (*P. salicina*), flowering cherry (*P. serrulata*), and Japanese apricot (*P. mume*). CVA is transmitted from plant to plant by grafting and vegetative propagation; no vector is known [2,4,5]. 

In cherry, CVA infection alone is usually latent and no disease can be related to the virus. However, in mixed infection with other viruses CVA can modulate the symptoms severity and affect scion/rootstock compatibility [8,9]. In contrast, symptoms of vein clearing, chlorosis, necrosis and mosaic were observed on CVA-infected apricot and myrobalan [10,11,12]. Although some of them may be potentially attributed to a mixed infection, no other viruses were detected by HTS in the CVA-infected apricot displaying vein-clearing symptoms [10]. 

LChV1 is a member of the genus *Velarivirus* in the family *Closteroviridae* [13]. The LChV1 genome is single-stranded positive-sense RNA of 16–17 kb and includes eight ORFs. The 5′-end is likely capped and the 3′-NCR is not polyadenylated and does not organize in a tRNA-like structure. Overlapping ORF1a and ORF1b encode replicase and RNA-dependent RNA polymerase (RdRp) and are translated as a polyprotein containing methyltransferase (MET), helicase (HEL), and RNA-dependent RNA polymerase (RdRp) domains. ORF2 encodes a small hydrophobic protein with a transmembrane domain. ORF3 encodes a homologue of the cellular HSP70h heat shock protein. ORF4 encodes a p61 protein with some similarity with the cellular HSP90. ORF5 and ORF6 code for the CP and minor capsid protein (CPm), respectively. Both CPs encapsidate the genome and are necessary for the cell-to-cell virus movement. ORF7 and ORF8 encode putative p21 and p27 proteins with no known functions [14,15,16,17]. 

LChV1 is a phloem-limited and graft-transmissible pathogen. There is no known insect vector for LChV1, so the virus spread among plants occurs mainly through vegetative propagation of infected plant material. The LChV1 hosts sweet and sour cherry and less frequently other *Prunus* species such as almond, apricot, peach, plum, and flowering cherry [18,19,20,21,22,23]. No clear symptoms are usually observed in non-cherry hosts. In contrast, in cherry LChV1 is associated with little cherry disease, which is distributed worldwide and manifested in the decreasing yield and fruit quality. Fruit symptoms occurring in susceptible sweet cherry cultivars include reduction in fruit size, color and taste while other cultivars can be symptomless. LChV1 is also responsible for Kwanzan stunting syndrome and Shirofugen stunt disease of flowering cherry [24,25]. 

The large collection of peach, nectarine, apricot, almond, plum, sweet and sour cherry genotypes, both local and introduced from North America, Southern Europe, and Central Asia, is maintained in the Nikita Botanical Gardens (NBG), Yalta, Russia [26]. An easy hybridization between *Prunus* species contributes to this gene pool, which is constantly being updated, expanded and exploited in breeding and biotechnological work. Many *Prunus* cultivars originated from this collection are distributed across the southern regions of Russia. Monitoring of viral diseases using various detection tools is important to limit the further spread of viruses. The phytosanitary status of this germplasm collection was studied using metatranscriptomic analysis of trees displaying virus-like symptoms on the leaves. The reads related to CVA and LChV1 were generated by HTS in two samples from *Prunus* interspecies hybrids. 

The objectives of this work were sequencing, assembly, and characterization of complete genomes of the Russian CVA and LChV1 isolates and their comparison with known isolates of these viruses available in GenBank. 

## 2. Results

### 2.1. Plant Material and Virus Detection

**Figure 1:**
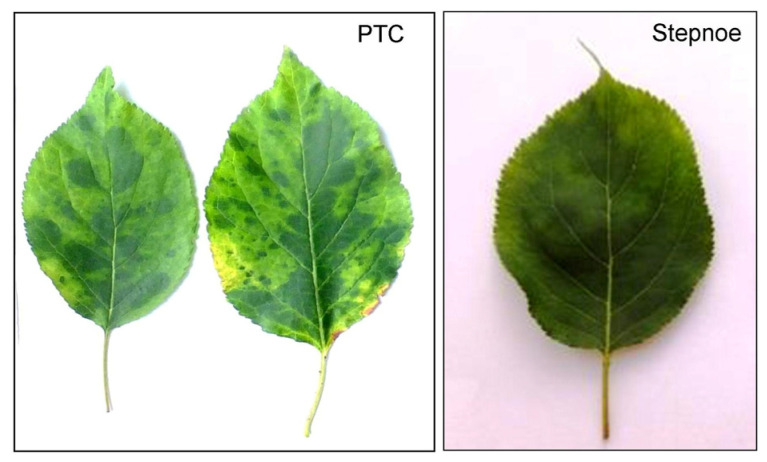
Virus-like symptoms on the leaves of *Prunus* trees infected with cherry virus A (PTC) and little cherry virus 1 (Stepnoe).

Two twelve-year-old *Prunus* trees displaying virus-like symptoms on the leaves (Figure 1) were selected for the metatranscriptomic analysis. These samples were named as follows: PTC from (*P. cerasifera* Ehrh. × *P. armeniaca* L. cultivar Shlor Tsiran) hybrid, grafted on a wild apricot, and Stepnoe from ((*P. cerasifera* Ehrh. × *P. armeniaca* L.) × *P. brigantiaca* Vill.) hybrid, grafted on *P. cerasifera*. 

The reverse transcription–polymerase chain reaction (RT-PCR) products of the expected sizes of 837 and 518 base pairs (bps) were obtained when analyzing total RNA from these specimens (Figure S1). Their sequencing by the Sanger method confirmed CVA and LChV1 in the corresponding samples. Thus, the HTS results were corroborated by the RT-PCR assay. 

### 2.2. HTS Results

The reads related to CVA and LChV1 were generated from the samples PTC and Stepnoe, respectively (Table 1). They covered the assembled virus-specific contigs with an average depth of coverage 400× to 720×. In addition, reads related to prunus necrotic ringspot virus (PNRSV) were revealed in the sample Stepnoe. 

### 2.3. Characterization of the CVA Genome

One contig 7426 nucleotides (nt) length was most closely related (99.5% identity) to the genome of the CVA isolates 19SP013 (MZ291922) and 13TF169_N11 (KY510919) from Canada as well as Ruzyne (ON088603) and WK (LN879388) from the Czech Republic and Australia, respectively. This contig covered the genomes of these isolates nearly completely and seemed to represent a new full-length CVA genome. With other CVA isolates, for which complete genomes were retrieved from GenBank, the isolate PTC shared 81 to 99% identity. Typically, of the genus *Capillovirus*, two ORFs were identified in the PTC genome. ORF1 of 7029 nt encodes a polyprotein of 2342 amino acid (aa) residues and ORF2 in a different reading frame 1392 nt long encodes the MP of 463 aa. MET, HEL, RdRp, MP and CP domains were mapped at positions 227–1153, 2552–3388, 3947–4900, 5506–6012, and 6632–7105, respectively. The 5′- and 3′-NCRs (excluding the poly (A) tail) consisted of 100 and 297 nt, respectively. The sequences of the 5′-terminal part of the MP gene, determined by HTS and Sanger sequencing, were identical. A near-complete genome of the Russian CVA isolate PTC was deposited in GenBank under accession number OQ865368. No other contigs showed similarity with any known virus available in GenBank. 

**Figure 2:**
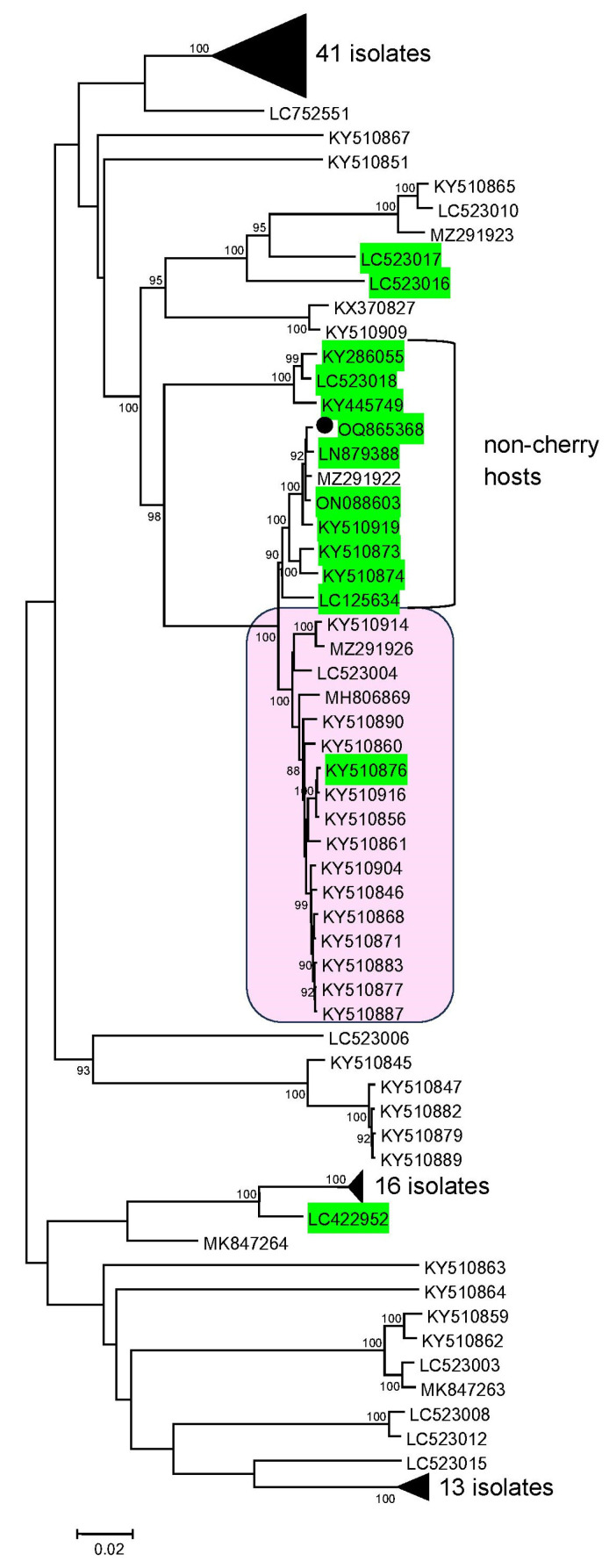
Phylogenetic analysis of cherry virus A (CVA) complete genomes conducted in MEGA7 [27]. The evolutionary history was inferred using the neighbor-joining method. The evolutionary distances were computed using the Kimura 2-parameter model. The accession numbers of isolates in GenBank are shown next to the end of branches. Bootstrap values (>85%) from 1000 replicates are shown next to the corresponding nodes. Non-cherry isolates are highlighted in green. Russian CVA isolate is indicated with a black circle (●). Pink filled clade unites cherry isolates potentially recognized with CVA-specific primers designed in this work. The scale bar indicates the number of substitutions per nucleotide. The black triangle means the condensed clade.

Phylogenetic analysis of all available full-length CVA genomes (*n* = 127, accessed on April 2023) showed that most isolates were clustered in several phylogroups (Figure 2). The PTC was assigned to the clade formed by two Canadian (MZ291922, KY510919), Czech (ON088603), and Australian (LN879388) isolates, thus confirming the results of sequence identity analysis. 

Both this and the sister clade mainly consist of the isolates from non-cherry hosts such as *P. armeniaca* (LC523018, KY510873, LC125634), *P. mume* (KY286055, KY445749, KY510874), *P. cerasifera* (ON088603, LN879388), and *P. salicina* (KY510919). Existence of a discrete cluster consisting of non-cherry isolates was in line with the previous results [9,12,28]. At the same time, a number of CVA isolates from plum (LC523016) and apricot (LC422952, LC523017, KY510876, KY510875, KY510880—the latter two are in the condensed clades) from Canada, Australia and India clustered with cherry isolates. 

CVA is known to be a genetically diverse virus and identity between complete genomes ranges 79% to 99% [4,12,28]. Given the considerable variability of CVA, virus-specific primers based on the full-length genome of the Russian isolate PTC were designed in this work. PCR with these primers enabled confirmation of the HTS results (Figure S1). In addition, in silico analysis of the alignment of the complete CVA genomes showed that, although non-universal, these primers would potentially recognize most (if not all) non-cherry isolates as well as cherry isolates grouped in the neighboring clade (highlighted in pink fill in Figure 2). Zero or one mismatch between forward and reverse primers and targeted genome sequences were found. At the same time, non-cherry isolates from other clades showed several mismatches with both forward and reverse primers that can crucially affect primer binding.

### 2.4. Characterization of the LChV1 Genome

BLASTn showed that a contig of 16,930 nt was most closely related (77.3% identity) to the genome of the Greece LChV1 isolate G15_3 (LN794218) from sweet cherry [29] and covered it nearly completely. Apparently, this contig represented the new full-length LChV1 genome. 

Typically, for the genus *Velarivirus*, eight ORFs were identified in the genome of the Russian isolate Stepnoe. ORF1a and ORF1b overlapped due to the ribosomal slippage at position 6931. All other ORFs were separated from one another by non-coding intergenic sequences ranging 1 to 171 nt in length. The MET, HEL, and RdRp motifs were predicted in the ORF1a/ORF1b-encoded polyprotein at positions 679–993, 2006–2263, and 2477–2744, respectively. CDD search showed that the product encoded by ORF4 was a viral homolog of the heat shock protein HSP90. 

The ORFs of the isolates Stepnoe and G15_3 were compared (Table 2). The ORF finder showed that ORF2, ORF4, and ORF6 to ORF8 of two isolates had the same length. The variability was rather randomly distributed along the genomes. ORF2 and ORF8 were most closely and, correspondingly, most distantly related on both the nt and aa levels. In ORF5, ORF6, and ORF8 the differences were more pronounced at the aa level, suggesting that some mutations were non-synonymous. In the isolate G15_3, ORF2 and ORF3 overlapped by eight nt, while in the isolate Stepnoe they were separated by an intergenic region of 4 nt in length. ORF3 and ORF5 of the isolate Stepnoe were shorter then their G15_3 counterparts, since in the latter the starts of the translation were shifted 12 nt upstream. Several indels were also detected when comparing these isolates. The Stepnoe ORF1a/b was shorter due to a 15 nt deletion of about six hundred nt upstream the MET domain. The intergenic region between ORF4 and ORF5 was shorter in the G15_3 due to a ten nt deletion. Although genomes of both isolates were coterminal, the G15_3 3′-NCR was shorter due to a 56 nt deletion. 

The genome regions of the isolate Stepnoe, which differed most strongly from the G15_3, were re-sequenced by the Sanger method using primers designed according to the full-length genome sequence of the Russian LChV1 isolate (Table S1). The sequences determined by the Sanger method were identical to those obtained by HTS. All indels and mismatches were confirmed by Sanger sequencing. The full-length genome of the Russian LChV1 isolate Stepnoe was deposited in GenBank under accession number OR260412. 

**Figure 3:**
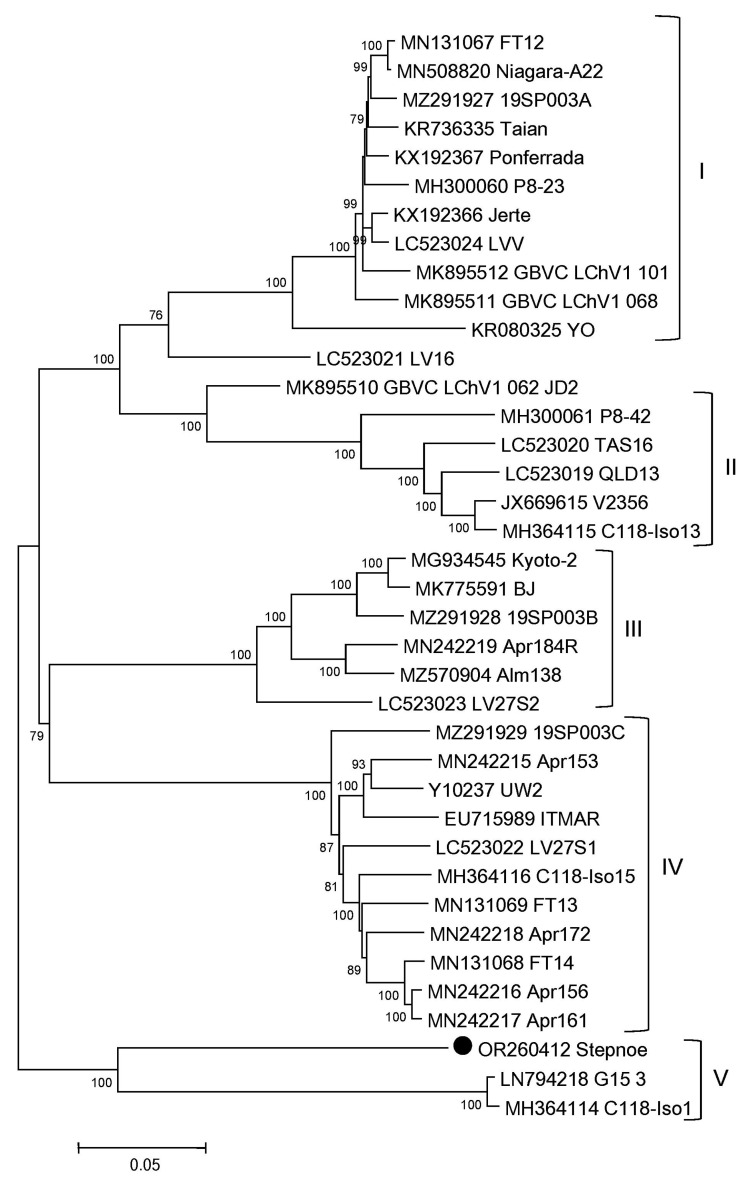
Phylogenetic analysis of little cherry virus 1 complete genome sequences. The tree was reconstructed using the neighbor-joining algorithm implemented in MEGA7. Bootstrap values (from 1000 replicates) are indicated next to the corresponding nodes as a percentage (>75%). Each of the five phylogroups (I–V) is joined by brackets. The accession numbers and names of isolates are shown at the end of branches. Russian isolate Stepnoe is highlighted with a black circle (●).

Phylogenetic analysis of all available full-length LChV1 genomes (*n* = 38, accessed on April 2023) showed that most isolates were clustered into five distinct groups (Figure 3). The isolate Stepnoe was assigned to the group V formed by two Greece isolates from sweet cherry [29], confirming the BLASTn results. The intragroup sequence identities ranged from 92.3% (group II) to 96.0% (group I). The complete genomes of the isolates G15_3 and C118-Iso1 in the group V shared 99.3% identity. The average intergroup identity was 76.8%.

## 3. Discussion

Both CVA and LChV1 were found on many stone fruit crops worldwide, but have never been revealed in Russia. In this study CVA and LChV1 were first reported from Russia, expanding the information on their geographical distribution. The viruses were detected by metagenomic HTS and confirmed using RT-PCR assay (Figure S1). 

CVA was found in the *P. cerasifera* × *P. armeniaca* interspecies hybrid showing virus-like symptoms on the leaves (Figure 1). This is consistent with the data that both myrobalan and apricot can be infected with CVA [10,11,12,30]. However, the observed symptoms differed from those previously described on these cultures, suggesting that they may represent a special response of this *Prunus* genotype to the CVA infection. Mixed infection with an unidentified virus is unlikely because no other viruses were detected in this tree by HTS. 

LChV1 was detected in the ((*P. cerasifera* Ehrh. × *P. armeniaca* L.) × *P. brigantiaca* Vill.) trihybrid. This suggests that myrobalan (*P. cerasifera*) and alpen plum (*P. brigantiaca*) can be potentially infected with this virus. Apricot is known to be the LChV1 host [18,19]. Interestingly, only mild mosaic symptoms were observed on the leaves of this tree despite it also seeming to be infected with PNRSV. 

CVA and LChV1 were found in *Prunus* trees grafted on a wild apricot and *P. cerasifera*, respectively. Since no insect vector is known for either virus, these trees were most likely infected through the rootstocks, which were no longer available and could not be tested for the viruses.

HTS technology can detect viruses by metagenomic analysis of infected plants and allows assembly of the complete virus genomes. Using this approach, the full-length genomes of the Russian CVA and LChV1 isolates were sequenced. The genomes of both viruses were shown to be typical for the *Capillovirus* and *Velarivirus* genera. Their positions among other isolates were determined using sequence identity and phylogenetic analyses. 

Phylogeny of all available full-length CVA genomes showed that, in agreement with the previous data [4,9,12,28,31], most isolates were clustered in several distinct phylogroups. Existence of a discrete phylogenetic group of non-cherry isolates is proposed to stem from the agricultural practice as cherry species are rarely grafted on non-cherry species thus preventing transmission of CVA isolates between these groups of hosts [9]. It seems logical that the isolate PTC from the interspecies hybrid of apricot and cherry plum was grouped together with non-cherry isolates.

Phylogenetic analysis of all available full-length LChV1 genomes showed that most isolates were clustered into five phylogroups (Figure 3). This result was in compliance with the previous phylogenetic data obtained by analyzing a smaller number of the complete LChV1 genomes [4,22,29]. The isolate Stepnoe was clustered with two Greece isolates from sweet cherry [29], thus expanding this divergent group. At the same time, the intragroup diversity among LChV1 isolates is known to be relatively low (3.3% to 7.4% at the nt level), whereas intergroup diversity is higher (15% to 39%) [29]. The intragroup complete genome identities calculated in this work ranged 92.3% to 96.0%, while the average intergroup identity was 76.8%. The isolate Stepnoe differed from its closest relative G13_3 considerably (Table 2). Their complete genomes shared only 77.3% identity. This obviously exceeds the level of intragroup variability usual for LChV1. Thus, the differences between the isolates G15_3 and Stepnoe are more correlated with intergroup diversity. It cannot be ruled out that the highly divergent isolate Stepnoe is the only representative of one more phylogroup of this virus so far. In this regard it is worth noting that the isolate Kyoto-2 (MG934545) was also the only member of the phylogroup G5 until recently [22,29]. However, the phylogenetic analysis of all currently available, full-length LChV1 genomes performed in this work showed that the Kyoto-2 is a member of the group III composed from new isolates, which have been sequenced in the very last years. 

In conclusion, trees infected with CVA and LChV1 were revealed in the NBG stone fruits collection. The full-length genomes of these viruses were sequenced for the first time in Russia and their positions among other CVA and LChV1 isolates from different hosts and geographical locations were determined. The prevalence of CVA and LChV1 in the collection and their genetic diversity have yet to be studied.

## 4. Materials and Methods

### 4.1. Sampling

Leaves displaying virus-like symptoms were gathered in the *Prunus* germplasm collection of the NBG in August of 2020. Individual samples composed of four to six symptomatic leaves were taken from each selected tree. The bagged samples were delivered to the virology department of Lomonosov Moscow State University and stored at 4 °C until used for the total RNA extraction. 

### 4.2. High-Throughput Sequencing (HTS)

Total RNA was extracted from fresh leaves using the cetyltrimethylammonium bromide (CTAB)-based protocol [32]. DNA libraries were synthesized using the TruSeq Stranded Total RNA Library Prep Plant kit (Illumina, San Diego, CA, USA) and sequenced on the Illumina MiSeq platform. Raw pair-ended reads of 250 bps were subjected to quality filtering and adapter removal using FastQC v.0.11.9 and Trim Galore v.0.6.5 (https://www.bioinformatics.babraham.ac.uk/projects/trim_galore (accessed on 19 September 2021)) using default parameters. Contigs were assembled de novo using the metaSPAdes program version 3.15 [33]. Virus-related contigs were identified by a BLASTn (https://blast.ncbi.nlm.nih.gov/Blast.cgi) against the GenBank nucleotide collection (accessed on 19 December 2022). The clean reads were mapped to the contigs using Bowtie2 v.2.4.4 [34]. The raw reads were deposited in the NCBI Sequence Read Archive (SRA) (https://www.ncbi.nlm.nih.gov/sra/PRJNA966926 (accessed on 19 September 2021)). The full-length genomes of the Russian CVA and LChV1 isolates were deposited in GenBank under accession numbers OQ865368 and OR260412, respectively. 

### 4.3. Sequence Analyses

To analyze the whole genomes of the Russian CVA and LChV1 isolates, the available sequences of these viruses were retrieved from GenBank. Multiple alignments of nt sequences, calculation of sequence identities, and phylogenetic analysis were performed in MEGA7 [27]. Phylogenetic trees were reconstructed using the neighbor-joining method and the Kimura 2-parameter model. ORFs in the complete virus genomes were identified using the ORF finder (https://ncbi.nlm.nih.gov/orffinder (accessed on 19 December 2022)). Conserved domains in virus proteins were mapped using the Conserved Domain Database (CDD, https://ncbi.nlm.nih.gov/Structure/cdd/wrpsb.cgi (accessed on 19 December 2022)). 

### 4.4. Reverse Transcription-Polymerase Chain Reaction (RT-PCR)

Total RNA, extracted as described above (see Section 4.2), was used as the template for the RT-PCR assay of CVA and LChV1. Random hexamer primers and Moloney murine leukemia virus (MMLV) reverse transcriptase (Evrogen, Moscow, Russia) were used for the first-strand cDNA synthesis. 

For the CVA detection, PCR was conducted using proof-reading Encyclo DNA polymerase (Evrogen) and primers CVA-F1 (5′-AGAATCAGGCTCTGTCTTAGGT-3′) and CVA-R1 (5′-TCTAGCTCTTGTTGAGCGTGGT-3′). These primers were designed based on the full-length genomic RNA of the Russian CVA isolate and amplified the 5′-terminal half of the *MP* gene generating a PCR product of 837 bps. The cycling conditions were 94 °C for 3 min, 35 cycles of 94 °C for 30 s, 54 °C for 30 s, 72 °C for 1 min, and a final extension at 72 °C for 10 min. 

For the LChV1 detection, PCR was also conducted using Encyclo DNA polymerase (Evrogen) and primers lchv-F5 (5′-AGCTATACGTGTGAACGAGAGA-3′) and lchv-R5 (5′-ATCATCGCCAATGTCTAAGGCA-3′), which amplified the genome region encompassing the 3′-end of ORF4, the 5′-end of ORF5 and intergenic sequence between them (positions 11,735 to 12,253 in the genome of the isolate Stepnoe), generating a PCR product of 518 bps. These primers were designed based on the full-length genome RNA of the Russian LChV1 isolate. The cycling conditions were 94 °C for 3 min, 35 cycles of 94 °C for 30 s, 52 °C for 30 s, 72 °C for 40 s, and a final extension at 72 °C for 7 min. 

Total RNAs from *Prunus* plants, which were shown to be CVA- and LChV1-free by HTS, were negative controls. Amplicons were analyzed by 1.5% (*w*/*v*) agarose gel electrophoresis, visualized by ethidium bromide staining, and photographed under the gel documentation system MultiDoc-It (Analytik Jena US LLC, Upland, CA, USA). PCR products were purified from agarose gel using the BC022 Cleanup Standard kit (Evrogen) and directly sequenced using Evrogen facilities.

## Figures and Tables

**Table 1:** Results of high-throughput sequencing of *Prunus* samples.

Sample	Number of Clean Reads ^a^ Per a Sample	Virus Detected	Number of Virus-Specific Reads ^b^
Stepnoe	2,903,382	Little cherry virus 1	27,359
PTC	2,250,077	Cherry virus A	21,341

^a^ Pair-ended reads of 250 nucleotides. ^b^ Determined using Bowtie2 v.2.4.4.

**Table 2:** Comparison of open reading frames (ORF) of the little cherry virus 1 isolates Stepnoe and G15_3.

ORF Name	ORF Length (Nucleotide (nt)/Amino Acid (aa))		Identity (nt/aa), %
	Stepnoe	G15_3	
ORF1a	6891/2296	6906/2301	77.5/82.4
ORF1b	1549/515	1549/515	82.9/92.8
ORF2	96/31	96/31	84.4/87.1
ORF3	1656/551	1668/555	79.4/85.7
ORF4	1554/517	1554/517	77.4/79.5
ORF5	1215/404	1227/408	74.6/74.4
ORF6	1989/662	1989/662	74.5/72.3
ORF7	696/231	696/231	78.3/86.0
ORF8	720/239	720/239	73.4/73.2

## Data Availability

Sequencing data were deposited in SRA and GenBank, and their accession numbers are provided within the article.

## Supplementary Materials

The following supporting information can be downloaded at: https://www.mdpi.com/article/10.3390/plants12183295/s1, Figure S1: Agarose gel electrophoresis of PCR products generated by RT-PCR assay of PTC and Stepnoe samples using cherry virus A (CVA)- and little cherry virus 1 (LChV1)-specific primers developed in this work (lanes 1 and 2, respectively). The arrows to the right to the picture indicate PCR products of the corresponding size. Lane 3—isolate PTC tested with LChV1-specific primers. Lane 4—isolate Stepnoe tested with CVA-specific primers. M—GeneRuler 100 bp Plus DNA Ladder (Thermo Scientific, Waltham, MA, USA). Table S1: Primers developed for the Sanger sequencing of little cherry virus 1 genome regions.
