# Supplementary material for: First Report and Complete Genome Characterization of Cherry Virus A and Little Cherry Virus 1 from Russia
Source: Plants (Basel). 2023 Sep 18;12(18):3295. doi: 10.3390/plants12183295 (PMC10534684; doi:10.3390/plants12183295)
Supplement: Supplementary file 1 [file plants-12-03295-s001.zip › Table S1.pdf]

Table S1. Primers developed for the Sanger sequencing of little cherry virus 1 (LChV1) genome regions.

Primer name	Primer sequence, 5'-3'	Target[a]	PCR product, bp
lchv-F1<br>lchv-R1	ACCTCACTGTTGATCGCCGA<br>AGTGTCTCGTCAGAGTGCCA	ORF1a (1312-1521)	209
lchv-F2<br>lchv-R2	TGAGTTGAGTTATGAGCGTGGT<br>AGCCCTGAATTGGCAATCTTGA	ORF1a (4010-4190)	180
lchv-F3<br>lchv-R3	GATGGGATGGTTCGAGTCTCT<br>GGGACATCACTATGAATTAGAGA	ORF1a (5094-5887)	793
lchv-F4<br>lchv-R4	GAATCTGAAATAGAACGCTTAGT<br>CGATAATGATTCTATTTCCCTGT	ORF3 – ORF4 (10237-10608)	371
lchv-F5<br>lchv-R5	AGCTATACGTGTGAACGAGAGA<br>ATCATCGCCAATGTCTAAGGCA	ORF4 – ORF5 (11735-12253)	518
lchv-F6<br>lchv-R6	CTGTAGAAGATAGAAGGTCAGA<br>GAGTCAATTGCACACCTGAACT	ORF5 (12372-12774)	402
lchv-F7<br>lchv-R7	GTGAAGTTTGGAAGAATTCTGGT<br>CTACTTCGACTTCATCATCACT	ORF6 (14135-14656)	521
lchv-F8<br>lchv-R8	GTAAATGACCAATTACTCGGTGGT<br>TGCAACACAGGTGGTCTATCACA	ORF8 – 3'-UTR (16412-16907)	495

[a] Target genes and positions (in brackets) in the genome of LChV1 isolate Stepnoe are indicated.

ORF - open reading frame; UTR - untranslated region.
