# Supplementary figures and images for: First Report and Complete Genome Characterization of Cherry Virus A and Little Cherry Virus 1 from Russia
Source: Plants (Basel). 2023 Sep 18;12(18):3295. doi: 10.3390/plants12183295 (PMC10534684; doi:10.3390/plants12183295)

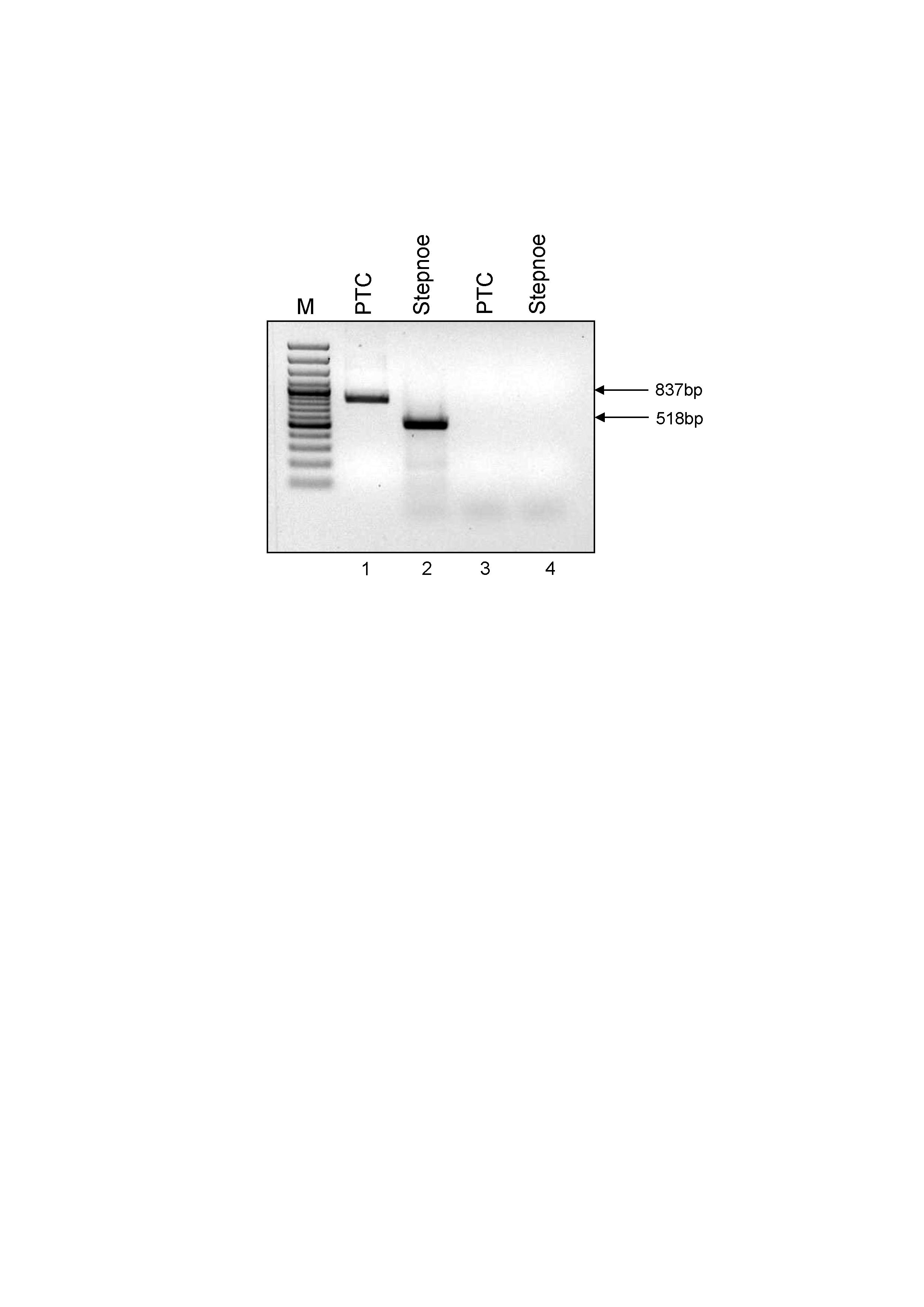

Supplement: Supplementary file 1 [file plants-12-03295-s001.zip › Figure S1.jpg]
