# Supplementary material for: First Report and Complete Genome Characterization of Cherry Virus A and Little Cherry Virus 1 from Russia
Source: Plants (Basel). 2023 Sep 18;12(18):3295. doi: 10.3390/plants12183295 (PMC10534684; doi:10.3390/plants12183295)
Supplement: Supplementary file 1 [file plants-12-03295-s001.zip › Table S1.doc]

**Table S1.** Primers developed for the Sanger sequencing of little cherry virus 1 (LChV1) genome regions.

| **Primer name** | **Primer sequence, 5'-3'** | **Target^a^** | **PCR product, bp** |
| --- | --- | --- | --- |
| lchv-F1 | ACCTCACTGTTGATCGCCGA | ORF1a (1312-1521) | 209 |
| lchv-R1 | AGTGTCTCGTCAGAGTGCCA |  |  |
| lchv-F2 | TGAGTTGAGTTATGAGCGTGGT | ORF1a (4010-4190) | 180 |
| lchv-R2 | AGCCCTGAATTGGCAATCTTGA |  |  |
| lchv-F3 | GATGGGATGGTTCGAGTCTCT | ORF1a (5094-5887) | 793 |
| lchv-R3 | GGGACATCACTATGAATTAGAGA |  |  |
| lchv-F4 | GAATCTGAAATAGAACGCTTAGT | ORF3 − ORF4 (10237-10608) | 371 |
| lchv-R4 | CGATAATGATTCTATTTCCCTGT |  |  |
| lchv-F5 | AGCTATACGTGTGAACGAGAGA | ORF4 − ORF5 (11735-12253) | 518 |
| lchv-R5 | ATCATCGCCAATGTCTAAGGCA |  |  |
| lchv-F6 | CTGTAGAAGATAGAAGGTCAGA | ORF5 (12372-12774) | 402 |
| lchv-R6 | GAGTCAATTGCACACCTGAACT |  |  |
| lchv-F7 | GTGAAGTTTGGAAGAATTCTGGT | ORF6 (14135-14656) | 521 |
| lchv-R7 | CTACTTCGACTTCATCATCACT |  |  |
| lchv-F8 | GTAAATGACCAATTACTCGGTGGT | ORF8 − 3'-UTR (16412-16907) | 495 |
| lchv-R8 | TGCAACACAGGTGGTCTATCACA |  |  |

^a^Target genes and positions (in brackets) in the genome of LChV1 isolate Stepnoe are indicated.

ORF - open reading frame; UTR - untranslated region.
